# Examination of tourists’ willingness to pay under different conservation scenarios; Evidence from reef manta ray snorkeling in Fiji

**DOI:** 10.1371/journal.pone.0198279

**Published:** 2018-08-01

**Authors:** Shannon E. Murphy, Ian Campbell, Joshua A. Drew

**Affiliations:** 1 Department of Ecology, Evolution and Environmental Biology, Columbia University, New York, NY, United States of America; 2 World Wide Fund for Nature, Global Shark and Ray Initiative Manager, WWF Pacific, Suva, Fiji; 3 Division of Vertebrate Zoology, Department of Ichthyology, American Museum of Natural History, New York, NY, United States of America; Department of Agriculture and Water Resources, AUSTRALIA

## Abstract

Wildlife-focused tourism is often considered as having the potential to play an integral part of threatened species conservation efforts, particularly through financial support. We focused on the direct financing of conservation by investigating tourists’ willingness to pay to snorkel with reef manta rays (*Mobula alfredi*) at Barefoot Manta, an ecotourism resort in the Yasawa group of islands in Fiji. Our results indicate that 82.4% of people surveyed would be willing to pay a mean value of ~ USD $9.2 (SE 0.9) more than the current cost, a 28% increase. Also, 89% of people surveyed would be willing to pay a mean value of ~ USD $10.2 (SE 0.9) more for a hypothetical scenario where they would snorkel with 50% fewer people, a 31% increase. We also investigated tourists’ willingness to make voluntary donations to the local community above an existing payment of ~ USD $10 that is built into the current snorkel payment of ~ USD $32.5. On average, 91.3% of the tourists interviewed were willing to donate additional funds with an average additional donation of ~ USD $8.6 (SE 0.5) to the community to pay for educational and environmental support, an 86% increase. There were few significant relationships between willingness to pay and demographic factors (including age, income, nationality, education, and others), suggesting that willingness to pay was widely held by the tourist population staying at Barefoot Manta Resort. Together, these results indicate that wildlife-based nature tourism could represent a potential, but not unlimited, income source to fund conservation in the Yasawa group, Fiji islands, and that conservation can arise from partnerships between local communities and the tourism sector.

## Introduction

Nature-based tourism, tourism largely centered around natural spaces, is a major economic driver, particularly in countries with high biodiversity [1]. Globally, marine nature-based tourism alone accounts for USD $47 billion annually [2] and has been integrated into the calculations of ecosystem services and values [3,4]. Wildlife-based tourism is a subset of nature-based tourism, which focuses on interactions between tourists and wild species. This form of tourism can provide a mechanism to support local conservation initiatives through financial support, non-financial contributions, socioeconomic incentives and education [5].

In coastal areas, one popular wildlife-based tourism option is snorkeling or scuba diving on coral reefs. For instance, on Australia’s Great Barrier Reef, tourism is valued at over USD $4 billion per year [6]. Snorkelers and scuba divers prefer healthy, protected reefs, and recreational divers are willing to pay more to dive within well-managed, limited-access fishery areas [7,8]. Marine wildlife-based tourism may also focus on close encounters with charismatic aquatic megafauna. For example, snorkeling with humpback whales (*Megaptera novaeangliae*) in Tonga generates over USD $5 million annually [9], while divers in the Maldives brought in USD $9.7 million in 2013 to swim with whale sharks (*Rhincodon typus*) [10]. Diving with sharks has been so profitable that it has helped spur several conservation plans including those in Australia [11], Palau [12], Fiji [13], and in French Polynesia, where each reef shark has been valued at over USD $2.5 million over its lifespan [14]. Using sharks as the centerpiece for wildlife-based tourism stands in stark contrast to using sharks for exploitative purposes, such as for their fins, and represent a way for local communities to capitalize on their local biodiversity in a more lasting and sustainable fashion [15]. While concerns exist about equity and distribution of funds among and within stakeholder groups [16], it appears that wildlife-based tourism of marine megafauna has the potential to provide an alternative livelihood for local communities. On the other hand, individual-based calculations can be potentially problematic. Issues concerning the marginal value of individuals may lead to management plans that are economically rational but ultimately detrimental to the long-term sustainability of the population, or the ecosystem in question. [17].

While tourism focused on sharks has become a global industry, wildlife-based tourism based on the other subclass of Elasmobranch (skates and rays) is less developed. However, the largest species of this group, the reef manta ray (*Mobula alfredi sensu* [19]) and the giant oceanic manta ray (*Mobula birostris*.), have the potential to provide a lucrative economic draw owing to their size and spatially and temporally predictable aggregations [18, 19]. Globally, the manta ray tourism industry is estimated to be worth USD $140 million, although only ten countries contribute 93% of that total [20]. Given their circumtropical distribution [21] and preference for rocky and coral shore habitats, *M*. *alfredi* has the potential for increased tourism opportunities. These opportunities for sustainable engagement with manta populations come at a critical time as both species of manta ray are listed on the International Union for Conservation of Nature (IUCN) Red List as Vulnerable, owing to high levels of exploitation and low reproductive rate [22–24]. While mantas have been harvested for generations [25], they are facing increasing fisheries pressure due to increased demand in Southern China for their gill plates, which are purported to prevent various illnesses [26, 27]. They are also facing increased fishing pressure due to bycatch, where manta rays are inadvertently caught in fishing gear such as nets and lines [28]. Because of increasing threats to their populations, manta rays (and all other rays in the genus *Mobula*) are now listed under Appendix II of the Convention on International Trade in Endangered Species of Flora and Fauna (CITES), which means the countries that are Party to CITES are required to issue permits to export devil and manta rays (products sourced from them) only after demonstrating that this trade does not harm wild populations [29].

The economics of tourism are susceptible to a variety of forces, both occurring domestically and internationally [1]. Understanding the motivations for tourists to visit a specific site, and coupling those motivations to a price point that is economically viable, are critical procedures for generating long-term sustainable, wildlife-based tourism practices [30]. Commonly, a willingness to pay (WTP) survey methodology is used to evaluate the motivations and acceptable price points for tourists [31, 32], and a large body of literature supports the use of these techniques in evaluating the opportunity costs of conservation action within an area (e.g. [33–37] for individual examples and [31] for a synthesis). The essence of these arguments is that tourism entrance fees provide a stable source of income for the management of a park, with parks ideally being revenue neutral, or even income generating. In some cases, studies have even argued that entrance fees to protected areas are undervalued [38], suggesting that there is an economic disconnect between the tourists and the managers.

An additional, and related, economic facet of marine conservation in areas with strong local control of resources is the direct payment to local communities for the offset of fisheries action, a type of payment for ecosystem services [39]. In Fiji, where local communities maintain traditional subsistence fishing rights over specific areas (*qoliqoli*), these payments instead of fishing resources have been successfully used to help fund conservation [40]. In these cases, tourist-generated income is used to fund reserves through offsetting the opportunity costs incurred by local communities for not fishing. For example, in the Namena marine reserve in Fiji, the income generated for the community from scuba divers has been used to fund fish rangers and a village scholarship to support education for villagers [41]. Even here, however, lack of formal institutions and inequitable distribution of benefits among communities has led to challenges in managing this ecosystem [42].

In this study, we explore the WTP for tourists engaged in manta-based tourism in the Yasawa Islands group in The Republic of Fiji. Specifically, we investigate (1) if tourists are willing to pay more than the current listed price (~ USD $32.5); (2) whether tourists would be willing to pay more to go on an experience that had fewer snorkelers in the water; and (3) if tourists would be willing to make voluntary payments to local communities to support education and local environmental initiatives. We hypothesize that tourists visiting Barefoot Manta Lodge on the island of Drawaqa in the Yasawa group, Fiji Islands 1) will be willing to pay more than the existing ~ USD $32.5 charge to snorkel with manta rays; 2) tourists will be willing to pay more than the existing ~ USD $32.5 to have a manta ray experience with fewer snorkelers in the water, and 3) tourists will be willing to pay more than ~ USD $32.5 if they know that a portion of the fee will go directly to local community education and conservation.

## Materials and methods

Surveys were conducted at Barefoot Manta Lodge on the island of Drawaqa in the Yasawa group, Fiji Islands. Barefoot Manta is a midsized lodge servicing 7000 individuals a year and provides various nature-based tourism opportunities including snorkeling and scuba diving on the surrounding coral reefs. The lodge is next to a seasonal (May to October) aggregation of up to 40 *M*. *alfredi*, where visitors observe an average of three manta rays per trip. This aggregation of manta rays is found in a channel on the north end of Drawaqa Island. The channel is approximately 250 meters long, and 10 meters deep at high tide, and a strong current runs through the channel in a southwestern direction. The manta rays will swim against this current near the surface of the water where they filter feed. There is also a cleaning station in this passage, which is a patch of coral where cleaner fish (*Labroides dimidiatus*) will clean the manta rays of bacteria and parasites.

**Fig 1:**
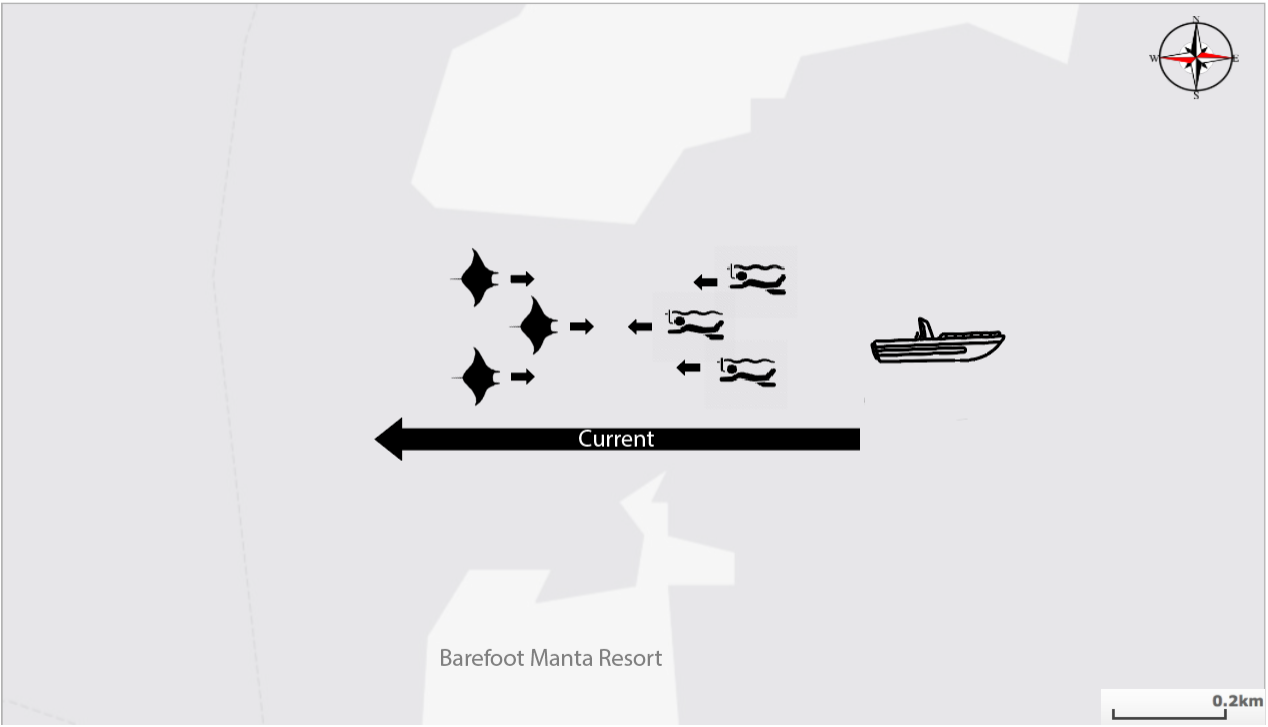
Schematic of manta dive operation at Drawaqa Island, Fiji. Swimmers are dropped in upstream (on the right) and float with the current, while the mantas typically swim up current (from left to right). After the drift swim, the snorkelers are picked up by boats past the channel (left).

This wildlife-based tourism encounter consists of guests entering the northeastern end of the channel, where they will disembark the boat and drift down through the passage, at which point they are picked up at the southwestern end of the channel by the lodge boat (Fig 1). Once retrieved by the lodge boat, they are brought back to the up current of the channel and begin to snorkel again. This cycle happens until the manta rays have left, or if people wish to return to the island. About five other resorts in the area conduct similar experiences, and each boat may have up to 10–20 snorkelers, bringing the total number of guests in the water during the daily high-tide encounter to about 70 at any one time.

Voluntary surveys were conducted from June-September of 2017 with guests at the lodge who expressed an interest in snorkeling with manta rays. WTP questions were asked in terms of Fijian Dollars (FJD), but have been converted to USD for this paper (conversion rate is USD 1 = ~FJD 2). All research was conducted with the permission of the lodge owner, the local community and with the approval of Columbia University (IRB approval permit number AAAR3782).

Both structured and open-ended interviews with guests were conducted during the pre-snorkel safety briefing. Before the briefing occurred, each participant was asked to disclose demographic information, including age, education level, nationality, ethnicity, annual household income, gender, previous snorkeling experience, previous experience in Fiji, and previous experience with ecotourism and wildlife-based tourism. The guests were asked about three aspects of their upcoming experience using a double-bounded, dichotomous contingent valuation survey [30]. At the time of the research, each manta dive cost ~ USD $32.5, and we asked guests if they were willing to pay ~ USD $5 more for a total of ~ USD $37.5. Next, we asked guests if they would be willing to pay ~USD $5 more for a total of ~ USD $37.5 if the number of guests per boat was halved from 20 to 10. Lastly, of the ~ USD $32.5 snorkel trip fee, approximately USD $10 is currently donated to the local community that are the traditional owners of the manta ray passage. We asked guests if they knew what programs were being funded by the community donation, and if they would voluntarily increase their payment up to ~ USD $12.5 more, effectively doubling the amount of money being paid to the local community.

Following the recommendations of Peters and Hawkins [30], positive responses about WTP were followed up with higher WTP price points (an additional ~ USD $7.5, $12.5, $17.5, $32.5, and $37.5). A double-bounded dichotomous contingent valuation method was used, so we were able to gain nuance from a negative response. For example, when a respondent offered a negative bid (e.g., they said they would be unwilling to pay ~ USD $5 more) we offered a lower bid (~ USD $2.5). This secondary lower bid allowed us to differentiate between negative responses about WTP due to philosophical differences (“I don’t think we should be giving money”) and negative responses due to economic differences ("I would be willing to pay $2.5 more but not $5 more"). This method is an important way of differentiating the underlying causes of negative results and provides more nuances into the analysis. For a complete record of the survey, please see S1 Table.

We aggregated the WTP data with unique individual identifiers. We then used a series of non-parametric tests to evaluate hypotheses about WTP. For binary (gender, previous experience in Fiji, previous snorkeling experience, previous experience with ecotourism and previous experience with wildlife-based tourism) categories, a Wilcoxon rank sum test was used. For comparisons including multiple categories (age, education level, nationality, ethnicity and annual household income), a Kruskal-Wallis rank sum test was used. We also used a Pearson correlation coefficient to look at the relationship between tourists’ previous experiences and WTP. To examine changes in WTP to the local community, we followed the same suite of statistical analyses.

To examine the relationship between price increase and tourists WTP, we calculated price saturation curves. These were generated for both scenarios (status quo and 50% reduction in tourists), using a generalized linear model and the function LT50 in the R package MASS [43]. We additionally calculated this for the sum that people would be willing to pay as an additional donation to the community. All tests were conducted in R [44] or PRISM [45]. All raw data from interviews, with identifying information removed, are presented in S2 Table.

## Results

We collected interviews from 85 individuals with summary data presented in Table 1 (for full results see S1 Table). Of these, 61 (72.8%) indicated that they would be willing to pay at least ~ USD $5 more to snorkel with manta rays (the current ecotourism scenario). Of the 24 individuals who indicated that they would not be willing to pay an additional ~ USD $5, nine (37.5%) indicated that they would be willing to pay an additional ~ USD $2.5 more; therefore, 15 (17.6%) individuals indicated that they would not be willing to pay any additional fee out of our total sample size of 85 individuals. In total, 70 (82.4%) people surveyed said they would be willing to pay more than the current cost, a mean value of ~ USD $9.2 (SE $0.9) and a 28% increase.

**Table 1:** Summary of demographic data regarding tourists interviewed about willingness to pay for the current ecotourism experience (Scenario 1), a reduced number of snorkelers in the water (Scenario 2), and voluntary payments to the local community (Scenario 3).

	Scenario 1	Scenario 2	Scenario 3
**Age Group**			
18–24	35	34	23
25–34	31	31	13
35–49	15	14	9
50–64	4	4	1
**Gender**			
Female	52	51	33
Male	33	32	13
**Annual Household Income**			
Low ($USD <30,000)	28	28	14
Medium ($USD 30,000–100,000)	23	22	11
High ($USD >100,000)	19	19	11
No Answer	15	14	10
**Education**			
No Schooling	1	1	1
Highschool	13	12	7
Some College	4	4	1
College	27	27	17
Graduate Degree	40	39	20
**Previously Visited Fiji?**			
Yes	13	13	8
No	72	70	38
**Previously Snorkeled?**			
Yes	81	79	42
No	4	4	4
**Previous Experience with Ecotourism?**			
Yes	45	43	27
No	40	40	19
**Previous Experience with Wildlife-Based Tourism?**			
Yes	40	38	24
No	45	45	22

Of the 83 individuals surveyed, 69 (83.1%) indicated that they would be willing to pay at least ~ USD $5 to hypothetically snorkel with ten people on the boat. Of the 14 individuals who would not be willing to pay an additional ~ USD $5, 5 (35.7%) indicated they would be willing to pay at least ~ USD $2.5 more; therefore, 11 (13.3%) individuals indicated that they would not be willing to pay any additional fee out of our total sample size of 83 individuals. In total, 74 (89.2%) people surveyed would be willing to pay a mean value of ~ USD $10.2 (SE 0.9) more for a hypothetical scenario where they would snorkel with 50% fewer people, a 31% increase.

Lastly, we surveyed 46 individuals about their willingness to voluntarily increase their donation to the community above the current ~ USD $10. When informed that these hypothetical increased donations would go to furthering "environmental or educational" opportunities in the community, we found that 42 (91.3%) of the tourist would be willing to give at least ~ USD $5 more. Of those who declined that increase, 0% suggested they would be willing to give ~ USD $2.5; therefore, 4 (8.7%) declined to give any additional funds to the community. In total, 42 (91.3%) of the tourists interviewed were willing to pay an additional ~ USD $8.6 (SE 0.5).to the community to pay for educational and environmental support, a 87.5% increase.

This study used double-bounded dichotomous contingent valuation to evaluate WTP. This methodology is robust because rather than simply offering tourists a “zero bid” (i.e., if they reject the opening bid of $5 then it is listed as a zero), we explore if the reason the opening bid is rejected is because 1) the interviewee simply does not want to pay any additional money or 2) the opening bid was too high. Indeed, when we look at the data, we found that 9 of the 24 “no” bids (37.5%) for the standard WTP study were because the opening bid was too high and that bid would have been accepted at ~ USD $2.5. Similarly, for the scenario with a reduced number of snorkelers, 5 of the 14 “no” bids (35.7%) would have been accepted at the ~ USD $2.5 level.

**Fig 2:**
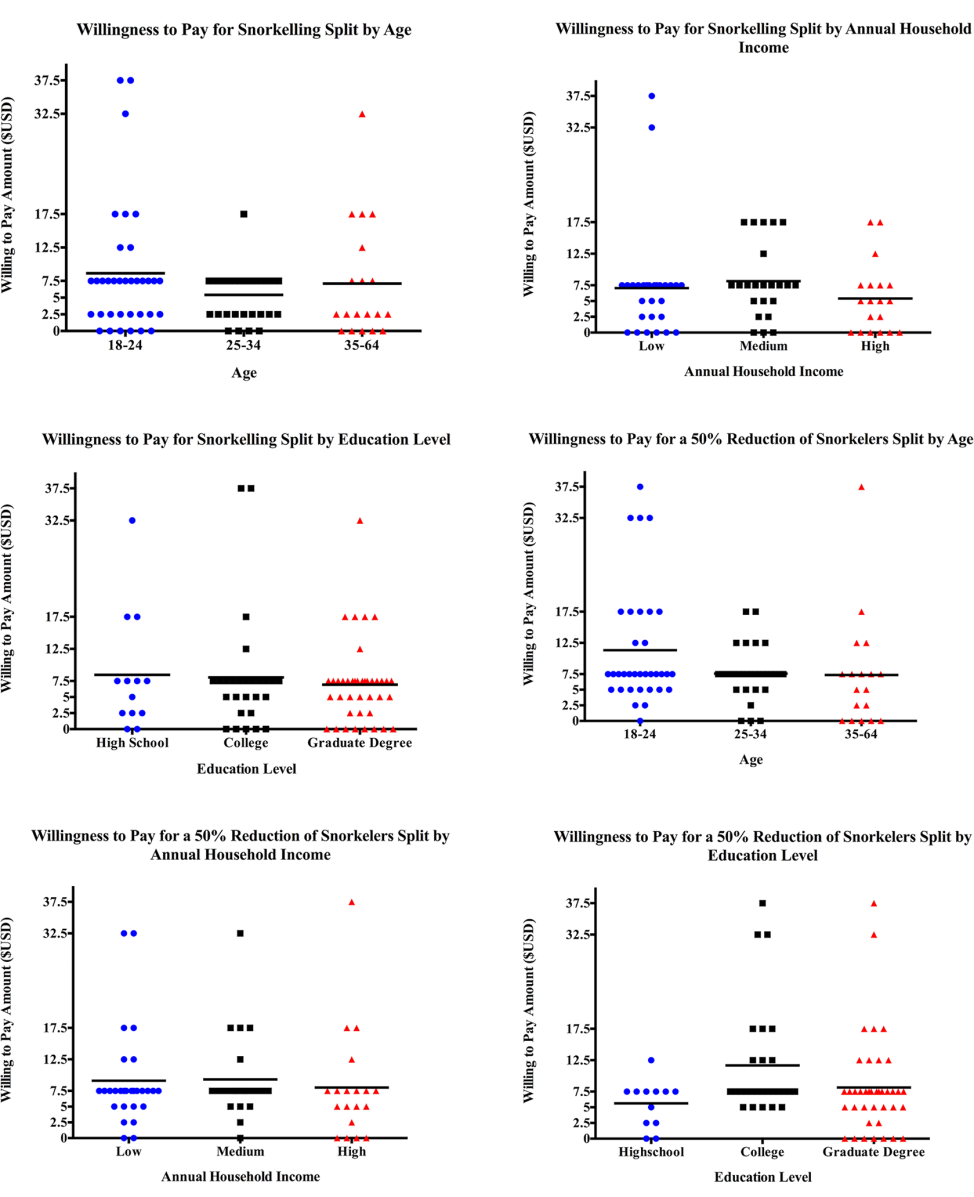
Tourists’ willingness to pay to dive with the current number of swimmers (apx. 20 swimmers per boat) under various socioeconomic factors: (a) Age, (b) Household Income, and (c) Education Level. Also, tourists’ willingness to pay to dive with a hypothetically reduced number of swimmers (10 swimmers per boat) under various socioeconomic factors: (d) Age, (e) Household Income, and (f) Education Level. Each dot represents a unique individual’s response and the black bars represent the mean willingness to pay value for each socioeconomic factor category.

**Fig 3:**
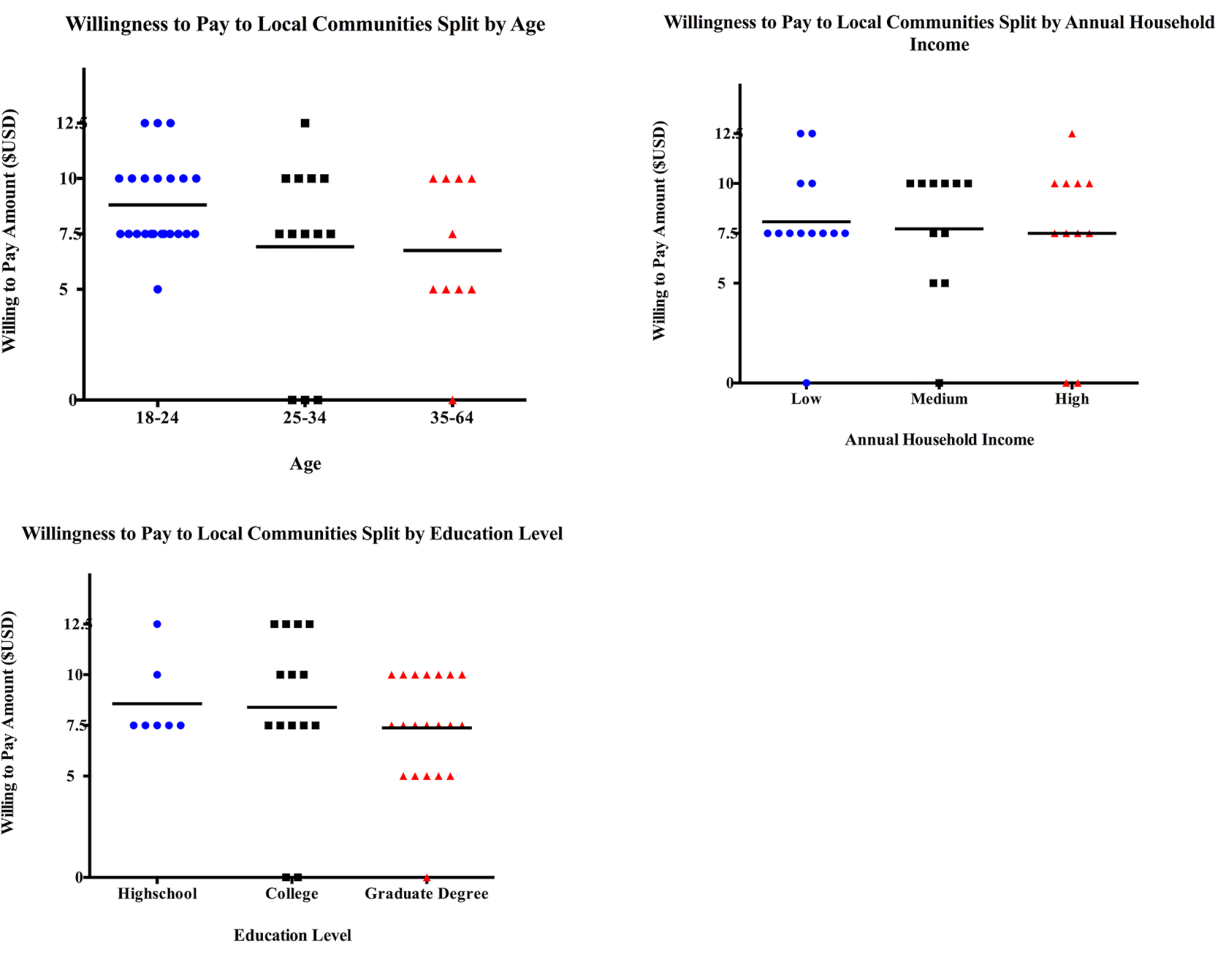
Tourists' willingness to donate to the local community for educational or environmental support under various socioeconomic factors: (a) Age, (b) Household Income, and (c) Education Level. Each dot represents a unique individual’s response and the black bars represent the mean willingness to pay value for each socioeconomic factor category.

The WTP was widespread across the population, with no significant associations among the WTP and age, education, nationality, ethnicity, annual household income, gender, or previous experience in Fiji for either the current scenario, (Table 1, Fig 2A, 2B and 2C), the reduced snorkeler scenario (Fig 2D and 2E), or for giving additional funds to the community (Figs 3A, 2B and 2C). There were significant interactions among the WTP between education and previous experience with ecotourism and previous experience with wildlife-based tourism (Table 2). There was a statistically significant association between education and WTP for fewer snorkelers (H = 6.45, p = .0398, Fig 2F), but there was no significance between groups when Dunn’s multiple comparisons test was run. Also, there was a statistically significant association between previous experience with ecotourism and WTP for fewer snorkelers, with those who have had previous exposure to ecotourism being willing to pay more to have 50% fewer snorkelers in the water (Mann-Whitney U = 579.5, p = .0071). Lastly, we found that those who did not have previous exposure to wildlife encounters would be willing to donate more to the local community (Mann Whitney U = 153.5, p = .0097).

**Table 2:** Results of various statistical associations among tourists willingness to pay under current situation (Scenario 1), with a reduced number of swimmers (Scenario 2), and to make donations to the community (Scenario 3). Significant values (α = .05) are in **Bold**.

	Test	Scenario 1	Scenario 2	Scenario 3
**Age**	Kruskal-Wallis	0.706	0.151	0.258
**Income**	Kruskal-Wallis	0.185	0.402	0.996
**Education**	Kruskal-Wallis	0.983	**0.004**	0.337
**Fiji**	Mann-Whitney	0.656	0.775	0.22
**Ecotourism**	Mann-Whitney	0.52	**0.007**	0.348
**Wildlife**	Mann-Whitney	0.509	0.082	**0.01**
**Gender**	Mann-Whitney	0.443	0.743	0.756

**Fig 4:**
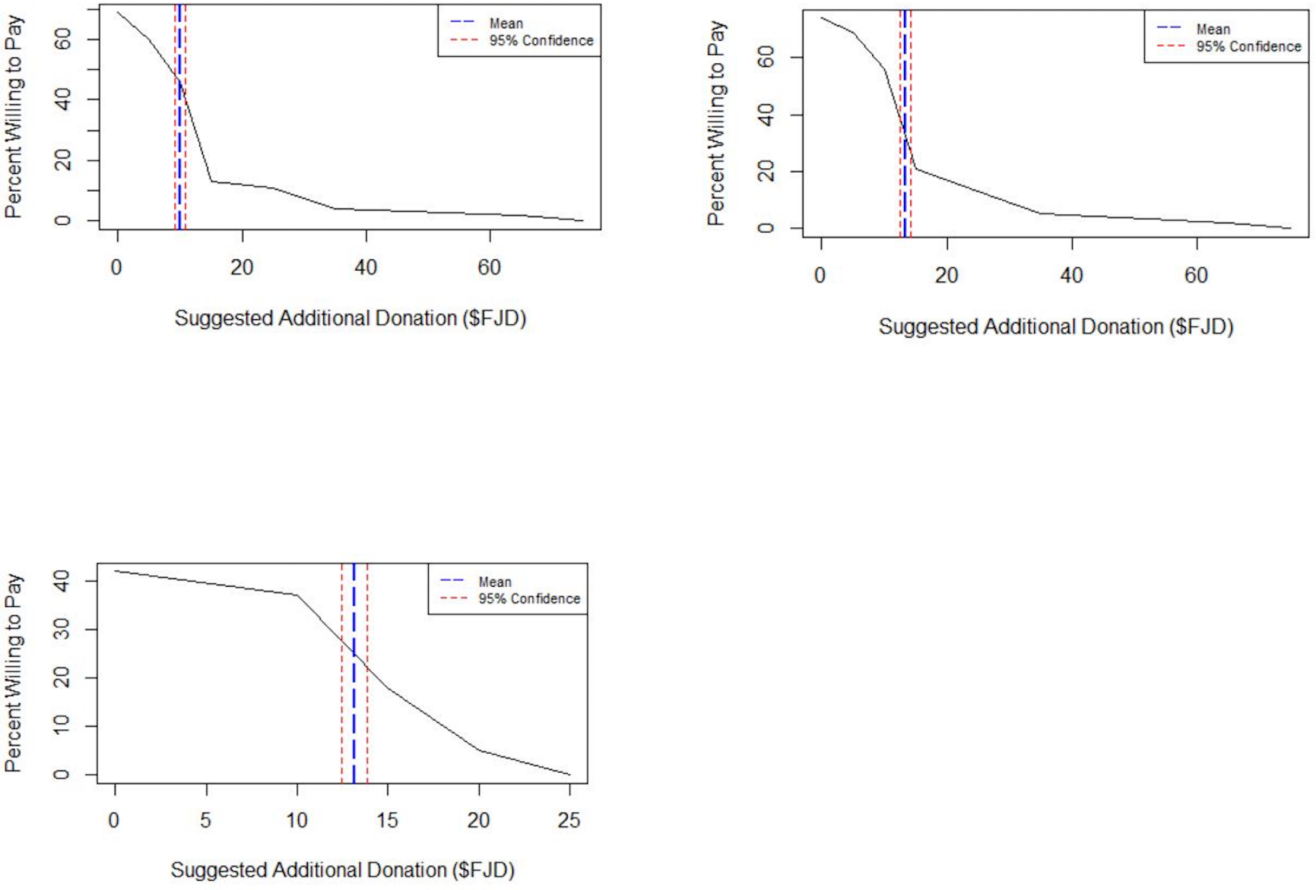
Response saturation curves for willingness to pay under the current ecotourism experience (a); for a reduced number of snorkelers in the water (b), and for voluntary payments to the community (c). The saturation curves reflect the total number of people willing to pay for a specific hypothetical increase above the current cost.

The saturation curves (Fig 4A, 4B and 4C) showed similar functions for WTP and WTP for a reduced number of tourists, however the price at which 50% of the people would be willing to pay was significantly greater for the reduced snorkeler scenario (~ USD $6.7, SE 0.4, Fig 4A) than for the status quo scenario (~ USD $5, SE 0.4; T-test, T = 2.899, p = 0.0042, Fig 4B). The 50% saturation price point for the amount tourists were willing to donate to the community was ~ USD $6.6 (SE 0.4, Fig 4C).

## Discussion

Results suggest that among a self-selecting group of tourists, there is a widely held consensus on the WTP additional fees to snorkel with manta rays, to snorkel with fewer people, and to make direct contributions to local communities to help foster further education in conservation. Moreover, these beliefs are consistent across much of the entire sampled population, with few significant differences among age, nationality, ethnicity, annual household income, gender, previous experience in Fiji and with snorkeling (Table 1). Where those significant differences did arise, they indicated that previous experience with ecotourism, and educational level, predicted an increase in WTP for a reduced amount of snorkelers. These results indicate people who have previous experience with ecotourism value fewer people during those experiences and have a preference for smaller, intimate and more ecologically friendly encounters [46]. Our data suggest that people who have had more ecotourism experiences can potentially draw on past experiences, and may have come to the conclusion that they value fewer people during an ecotourism experience. While education level indicates a change in WTP for fewer snorkelers, this relationship is unclear and further research will be needed to clearly define this relationship. Additionally, we found that exposure to wildlife-based tourism seemed to predict a decreased WTP to the local community. Concerns about making direct donations to communities are not uncommon [7], especially if it is unclear where additional funds are being used. However, the Barefoot Manta Lodge makes this information known to their guests before they snorkel with manta rays (33% to the resort, 33% to a manta ray conservation organization called Manta Trust, and 33% to the local communities that reside beside the channel). Since surveys were distributed to guests before they snorkeled with manta rays, they answered these questions before knowing what exactly their money would be going towards.

Manta rays are a global tourist attraction, with manta-based tourism occurring in the Maldives [47], Indonesia [48], Palau [12] and at least 20 other countries (see [20] for a global review). Our results reinforce these findings and expand on them by examining WTP across sectors, and by coupling payments for ecosystem services—we show broad-scale support for increased payments to snorkel with mantas. Barefoot Manta Resort has higher prices implemented for manta ray snorkeling (~ USD $32.5) compared to neighboring resorts such as Manta Ray Island Resort (~ USD $22.5); however, it is the only resort in the area that directly pays the local community for the use of the channel (~ USD $10), which is why the price is higher compared to other resorts. Thus, here we see tourists’ payment for direct extractive ecosystem services (e.g., watching the mantas) being coupled with community involvement and maintenance of these ecosystem services (e.g., knowing that the community has educational and environmental opportunities). By diversifying the payments for wildlife-based ecotourism, a greater proportion of the tourist funds can be captured by the local community–a key component to success in community-based ecotourism [49].

While tourist behavior in aggregate was largely supportive of payments for manta encounters, examination of the data reveals two nuances that provide more insight into the behavior of customers. First, by examining WTP saturation curves for manta ray snorkeling, we see that there are two different payment behaviors. Under both scenarios, approximately 80% of the population views the increase in proposed costs as an elastic cost–that is the greater the cost, the fewer people willing to pay it. This linear relationship, however, reaches an inflection point and, for the final 20% of the population, the cost for snorkeling with mantas appears much more inelastic (that is, a large increase in price is required to reduce people’s WTP). The curve for the donation to the community question does not display such an inflection point, and therefore, we can infer that such payments are price elastic. Wildlife tourism in general [50], and especially with marine megafauna, is typically considered price inelastic [51, 52]. Our results here may be due to a large proportion of “backpackers” coming to Drawaqa Island. Interview data suggest that many of the tourists want to see as many resorts along the island chain as possible and an increase in manta snorkeling prices may translate into fewer additional visits. Thus, while overall there is a WTP, when data are aggregated, variation within the tourists’ desires and behaviors will be hidden. We find an analogous example to our results from the air travel industry. Air travel is considered price elastic as a whole; however business class traversers are much less sensitive to changes in price than those flying economy [53].

Our results also show that there exists a potential pool of funding from tourists that could be applied directly to the community. The respondents were, on average, willing to increase their existing donation by almost 86%, provided that those additional funds went to education or environmental conservation by the village. While this represents a novel and reliable source of funding for the community, the fiduciary structure of the village could render these donations problematic. Customary *qoliqoli* owners are under no legal obligation to spend the dividends from their *qoliqoli* in any particular fashion. This lack of obligation could generate potential disconnects between the tourists and the community natural resource holders. Concerns about making direct donations to communities are not uncommon [7], and in some countries, have been ameliorated by an NGO acting as an intermediary body [8]. While this arrangement contains problematic neocolonial assumptions about the ‘capacity’ of local communities to manage their resources [54], the fact that donations to the community from tourists may represent an additional source of income may offer an additional income diversification beyond extractive activities on these reefs [49]. Finally, the WTP curve (Fig 3C) shows that this behavior is in part price elastic, and therefore communities, managers, and NGOs should be cautious when trying to estimate the magnitude of potential income for the community that tourism may provide.

When examining tourists’ WTP using a CV survey, it is important to keep in mind the potential biases that exist within this methodology. There may be “hypothetical bias” within these surveys, as all WTP are hypothetical scenarios and participants do not need to make real commitments based on their responses [55]. Individuals could choose a bid in theory, but not in practice, which could result in an increased bid choice. Also, there is the possibility of strategic bias, where participants may select a bid based on the assumption that new prices for snorkeling with manta rays will be implemented in the future [34]. This bias could have been reduced by informing participants beforehand that there is no intention of changing the price to snorkel with manta rays at this moment in time. While these biases do exist in CV surveys, this methodology ensures we can determine the maximum WTP for each participant, by offering a sequence of bids based on positive and negative opening bid responses [55].

What future might a tourism-based conservation program for manta rays look like in Fiji? There are already successful elasmobranch wildlife-based tourism operations in Fiji, including the Shark Reef Marine Reserve [13], positioning Fiji as a globally important destination for marine wildlife-based tourism. However, success in one area may set up expectations for other communities. Failures in community-based conservation initiatives arise when costs are accrued by the general population, while benefits are too dilute to be recognized at the individual level. This unequal distribution of benefits feeds into “conservation capture” by elites in the government or in the community [56]. Conversely, success in those community-based payment fees come when the benefits accrue across local communities, when those benefits are dependent on positive (and quantifiable) conservation outcomes, and when future conservation successes translate into additional benefits to local groups [56]. Here we show that the potential for direct contributions to the *qoliqoli* owners exists, but those owners are under no legal obligation to carry out conservation with those funds. Should there be a disconnect between perceived targets of compensation for ecosystem services and the realized expenditure of those funds, tourists could be left feeling less enthusiastic about future compensation schemes. For example, in Chile, there was a general increase in WTP for tourists to dive in a managed reef. However, interviews revealed that the tourists’ enthusiasm was tempered due to inefficiencies built into the system and an overall mistrust of how conservation funds will be distributed [7]. This could explain why we saw tourists were willing to pay on average more for scenarios that specifically stated where the money would go, such as paying money directly to the local community, rather than just asking if they would pay more to snorkel in general.

In the Anthropocene (the current epoch of unprecedented anthropogenic change *sensu* Moore [57]), tourism can feed off of narratives of loss. This “last chance” or “extinction” tourism can be important in motivating a segment of the tourist population to engage with endangered species or habitats [58]. For example, Piggott-McKellar and McNamara [59] found that tourists were coming to the Great Barrier Reef in large numbers in response to recent reports of massive bleaching [60], yet they downplayed the impacts of their tourism on the reef itself. Similarly, at our study site, there was little discussion either among the staff or the visitors about the potential negative impacts (other than direct contact) of tourism activities on the animals themselves or their ecosystem. This can include the use of flash photography and snorkelers diving towards the manta rays, which can force a change in their swimming direction and disrupt their feeding [60]. Behaviors like this can be hard to monitor since there are typically multiple boats in the channel, and these activities happen underwater [61]. While the resort itself has instituted several environmentally-friendly initiatives (coral replanting, dune conservation, etc.), and mandates a pre-dive briefing where basic manta biology and encounter safety are outlined, the link between the carbon footprints of the trip (including travel, boat fuel, etc.) was largely unexamined. Approximately 80% of the respondents (and two out of three authors) were from North America, and our qualitative data suggest the tourists’ impacts on the reef were often unstated or unexamined. Additionally, on a more fundamental scale, we show how capitalism can both drive threats to manta rays and simultaneously, through wildlife-based tourism, can offer potential conservation solutions [58].

The area off of Drawaqa island in the Yasawas, where the manta ray diving takes place, is a voluntary no-take reserve and the result of the local community and *qoliqoli* managers partnering with tourist resorts within the area. This combination has the benefits of being adaptive, community-based, and generating income. However, it also lacks formal legal representation as currently the Fijian government only recognizes the community’s right to fish in an area, not the right to own that area *per se*. This murky legal representation may limit the investment and enthusiasm of tourist resorts in these informally codified conservation measures. However, given the limited resources facing conservation NGOs and government agencies, the difficulty of enforcing distant reserves, and the absolute necessity of community buy-in, locally derived partnerships between communities and the tourism sector represent a novel approach to using capitalism to foster conservation.

## Supporting information

S1 Table Survey questionnaire for tourists at Barefoot Manta Resort. (DOCX)

S2 Table Questionnaire results from tourists at Barefoot Manta Resort. (CSV)
