# Supplementary material for: Examination of tourists’ willingness to pay under different conservation scenarios; Evidence from reef manta ray snorkeling in Fiji
Source: PLoS One. 2018 Aug 1;13(8):e0198279. doi: 10.1371/journal.pone.0198279 (PMC6070171; doi:10.1371/journal.pone.0198279)
Supplement: S1 Table — (DOCX) [file pone.0198279.s001.docx]

**Demographic data**

Age

Nationality

Gender

Snorkel before Y/N

Ecotourism before Y/N

Have you ever participated in a wildlife encounter ecotourism experience before

Been to Fiji before Y/N how many times?

**Before excursion:**

Currently the fee to snorkel with Mantas is $65 Fijian Dollars

Where do you think that money goes?

Would you be willing to pay 10 dollars more, for a total of $75 FJD to snorkel?

If Yes

15 dollars more to 80?

25 dollars more to 90?

35 dollars more to 100?

65 dollars more to 130?

If no

5 dollars more to $70

0 dollars more?

Why?

The total number of visitors on a typical Manta swim is 20. Given this do you feel this is an appropriate number of visitors?

If the number of visitors was reduced by 50% percent to 10 snorkelers, would you be willing to pay 10 dollars more, for a total of $75 FJD to snorkel?

If Yes

15 dollars more to 80 (a 23% increase in price)?

25 dollars more to 90 (a 38% increase in price)?

35 dollars more to 100 (a 53% increase in price)?

65 dollars more to 130 (a 100% increase in price)?

If no

5 dollars more (a 7% increase)?

0 dollars more?

Why?

**After excursion:**

You just had the chance to swim with Mantas, did you see Mantas today?

How would you rank your overall experience (0-10, 10 being highest)

Now we are going to ask you similar questions as before.

Would you be willing to pay 10 dollars more, for a total of $75 FJD to snorkel?

If Yes

15 dollars more to 80

25 dollars more to 90

35 dollars more to 100

65 dollars more to 130

If no

5 dollars more to $70

0 dollars more

Why?

The total number of visitors on a typical Manta dive is 20. Given your experiences on this excursion, do you feel this is an appropriate number of visitors? How many visitors did your boat have today?

If the number of visitors was reduced by 50% percent to 10, would you be willing to pay 10 dollars more, for a total of $75 FJD to snorkel?

If Yes

15 dollars more to 80 (a 23% increase in price)

25 dollars more to 90 (a 38% increase in price)

35 dollars more to 100 (a 53% increase in price)

65 dollars more to 130 (a 100% increase in price)

If no

5 dollars more (a 7% increase)

0 dollars more?

Why

Currently the local communities receive $0 from the resort charges

Would you be willing to pay an additional $15 FJD if you knew that fund was going to help support the local people, through education and environmental programs whose waters you swam in?

If Yes

$20 dollars

$25 dollars

$50 dollars

If no

$10 dollars

$5 dollars

$0 dollars

Why?
